# Fabrication of Hybrid Alginate Hydrogel Beads Reinforced with Activated Carbon and Evaluation of Their Potential for Controlled Eugenol Release

**DOI:** 10.3390/pharmaceutics18050598

**Published:** 2026-05-14

**Authors:** Kaan Karaoğlu, Mehtap Atak, Nuray Yılmaz Baran, Talat Baran

**Affiliations:** 1 Department of Chemistry and Chemical Processing Technologies, Vocational School of Technical Sciences, Recep Tayyip Erdoğan University, 53100 Rize, Turkey; kaan.karaoglu@erdogan.edu.tr; 2 Department of Medical Biochemistry, Faculty of Medicine, Recep Tayyip Erdogan University, 53200 Rize, Turkey; 3 Department of Chemistry, Faculty of Science and Letters, Aksaray University, 68100 Aksaray, Turkey; nybaran@aksaray.edu.tr (N.Y.B.); talatbaran@aksaray.edu.tr (T.B.)

**Keywords:** eugenol, controlled release, alginate hydrogel beads

## Abstract

**Background/Objectives**: This study presents the development of an activated carbon/sodium alginate-based gastric-retentive delivery system aimed at enhancing the gastroprotective efficacy of eugenol (Eug) in simulated body fluids. **Methods**: Hybrid hydrogel beads were fabricated using tea waste-derived activated carbon (AC) as a core material and sodium alginate as a wall material. **Results**: The system achieved a loading capacity of 3.37 ± 0.11 mg Eug/g hydrogel beads, and in vitro assays revealed a controlled release profile, with cumulative release reaching 0.694 ± 0.006 mg/g hydrogel beads in simulated gastric fluid (SGF) and 0.198 ± 0.002 mg Eug/g hydrogel beads in simulated intestinal fluid (SIF). **Conclusions**: Kinetic modeling confirmed a predominantly diffusion-controlled process with non-Fickian transport mechanism, indicating combined diffusion and matrix relaxation. By maintaining local therapeutic concentrations in the gastric mucosa, this pH-responsive Alg/Eug@AC system offers a sustainable strategy to overcome Eug’s low bioavailability and provide effective gastroprotection against oxidative damage.

## 1. Introduction

Eugenol (Eug, 4-allyl-2-methoxyphenol), the main ingredient of clove essential oil is a phenolic monoterpenoid with a low molecular weight and a moderately lipophilic nature [1]. Eugenol has a rich historical background of therapeutic uses because of its anti-inflammatory [1], antibacterial [2], antioxidant [3] properties. With its powerful antioxidant properties resulting from donation of electrons from hydroxyl group [4], Eug directly scavenges free radicals, limiting the formation of reactive oxygen species (ROS) and reactive nitrogen species (RNS). This effect contributes to the protection of DNA and proteins from oxidative damage at the cellular level [5]. Additionally, various studies have shown that Eug suppresses the expression of proinflammatory cytokines such as interleukin-1β (IL–1β) and interleukin-6 (IL–6), tumor necrosis factor alpha (TNF-α), and prostaglandin E2 (PGE2), as well as the inducible nitric oxide synthase (iNOS) and cyclooxygenase–2 (COX–2), and can modulate certain cellular signaling pathways through the activation of the leukotriene C4 and 5-lipoxygenase (5-LOX) pathways [6,7,8]. Moreover, Eug can be employed as bioenhancer to increase efficacy of the drugs via modulation on metabolic pathways [9,10,11].

Due to its lipophilic properties, Eug has low solubility in water, and studies conducted on rats have shown that its oral bioavailability is low (4.25%) [12]. Studies have also shown that Eug degrades easily due to environmental conditions such as temperature, oxidation, light, and humidity [13] as well as disadvantages such as rapid absorption from the stomach and small intestine, the development of new transport systems is of interest for controlled release of Eug. Recent studies have demonstrated that encapsulating Eug within polymeric micro/nanocarriers, lipid-based systems, or emulsion structures increases chemical stability, reduces volatility and oxidative degradation, and provides a more stable release profile [13,14,15,16,17].

In particular, targeted and controlled release strategies to the gastric environment may contribute to maintaining local therapeutic concentrations in the gastric mucosa and limiting potential irritant/toxic effects associated with excessive exposure, supporting the gastroprotective effects of Eug at low doses reported in the literature [18,19,20]. In a previous work by Longo et al., it was reported that while the 100 mg/kg dose aggravated ulcers at 7 days and failed to promote healing at 14 days, the 1 mg/kg dose accelerated gastric healing at both intervals [19]. Furthermore, the highly porous architecture of such activated carbon materials makes them excellent candidates for biomedical applications, particularly as versatile matrices for the loading and controlled release of active pharmaceuticals [21]. Tea waste residue represents an exceptionally promising lignocellulosic biomass precursor, inherently suited for the development of micro- and mesoporous structures. Beyond its structural potential, its global abundance and cost-effectiveness make it an ideal candidate for large-scale environmental applications [22,23]. In light of these findings, the aim of this work was to establish strategies for the development of a gastric retentive drug delivery system to ensure the sustained release of this effective low dose of eugenol within the gastric environment. To achieve this goal, we have designed a two-component hydrogel-controlled release system using activated carbon as the core material for Eug deposition, and sodium alginate as the wall material for encapsulation as well as for modulation of pH-dependent Eug release.

## 2. Experimental

### 2.1. Equipment

A Quantachrome BET analyzer (Quantachrome Instruments, Boynton Beach, FL, USA) was used to evaluate surface properties of the activated carbon material. AC sample was degassed under vacuum at 105 °C for 12 h prior to the measurement. Brunauer–Emmett–Teller (BET) and BJH (Barrett, Joyner, and Halenda), QSDFT (quenched solid density functional theory) methods were used to estimate the effective surface area of the material. Both the adsorption profile of the Eug to activated carbon and the desorption profile from hybrid carrier were evaluated by HPLC. HPLC analyses were conducted using a Shimadzu LC–20AT HPLC (Shimadzu Corporation, Kyoto, Japan) instrument with a photodiode array detector (PDA) and a GL Science InertSustain C18 reverse phase column (GL Sciences Inc., Tokyo, Japan) (10 μm particles, 200 × 4.6 mm I.D.) with a column temperature of 30 °C. Acetonitrile/water containing 0.1% trifluoroacetic acid (62:8 *v*/*v*) was used as the mobile phase and the flow rate was 0.45 mL/min. Eugenol content of aqueous solutions was analyzed in triplicate HPLC injections and results were given as $\bar{X}\pm SD$; mean ± standard deviation.

### 2.2. Fabrication of Alginate-Coated Activated Carbon Hydrogel Beads (Alg/Eug@AC)

AC was obtained from carbonization of industrial tea processing wastes including the discarded leaves, stems, and buds of tea plants. In total, 50.0 g of waste was milled using a laboratory-type blender and sieved using a 40-mesh sieve cascade. Then 10 g of waste was loaded in a 500 mL beaker with 125 mL distilled water and 28 mL H_3_PO_4_ (85%) as the activation agent was heated to 70 °C for 7 h with continuous stirring. Slurry tea waste was separated by filtration and loaded in a ceramic boat for carbonization in a tube furnace operated at 800 °C under argon atmosphere (100 mL min^−1^ flow rate) using a heating rate of 10 °C min^−1^ [24]. AC material, then, was cooled to room temperature and washed with 100 mL distilled water three times.

Eug adsorption on AC (Eug@AC) was achieved via two different strategies. First, an ethanolic solution of Eug ranging from 10 ppm to 500 ppm was added onto 0.15 g of AC in a beaker and stirred using a magnetic stirrer for 4 h. The suspension was separated by centrifugation at 4000 rpm for 10 min and the solution was kept for HPLC analysis. Second, 0.1 g of pure Eug was added on to the AC and mixed with a glass rod for 5 min. Subsequently, 0.3 g sodium alginate and Eug@AC were added to a beaker with continuous stirring. After adding 40 mL of water to the beaker, the mixture was subjected to homogenization with a high-speed laboratory homogenizer (Wise Tis, DAIHAN Scientific Co., Ltd., Wonju, Republic of Korea). The mixture was transferred into a burette and added to a 100 mL (0.03 M) CaCl_2_ solution dropwise within 10 min. Finally, the hydrogel beads were filtered over filter paper and washed with 200 mL of distilled water, and stored for 24 h in a refrigerator at 4 °C.

### 2.3. Eugenol Release from Alginate-Coated Activated Carbon Hydrogel Beads (Alg/Eug@AC)

Eugenol-release profile of the Alg/Eug@AC beads was investigated both in simulated gastric fluid (SGF) and simulated SIF by HPLC at 22 ± 3 °C. Simulated SGF contains aqueous solution of 25.0 mM sodium bicarbonate, 6.9 mM potassium chloride, 47.2 mM sodium chloride, and 0.1 mM magnesium chloride. Then, 36 mL of preheated pepsin (2000 units/mL) and 150 µL calcium chloride (0.15 M) were added to the solution before it was acidified to pH 3.0 with 1.0 N HCl. Simulated SIF consists of 85 mM sodium bicarbonate, 38.4 mM sodium chloride, 0.8 mM potassium dihydrogenphosphate, 6.8 mM potassium chloride 0.33 mM magnesium chloride, and 6.0 g pancreatin. The pH of the SIF was adjusted to 7.0 by using 1.0 N NaOH and a final volume of 50 mL was obtained by adding deionized water. [25].

To evaluate Eug-release capacity of Alg/Eug@AC beads, 15 mL of SGF transferred into a falcon tube then 25.0 g of Alg/Eug@AC was added to the tube. The tube was placed on a roller mixer at 22 ± 3 °C at a constant speed of 50 rpm, then the liquid phase was transferred into a tube at 30 min intervals for 2 h, and then fresh 15 mL of SGF was added to the tube for the next cycle. A transferred aqueous sample was diluted two-fold by adding acetonitrile and taken into an HPLC vial. After the last cycle, Alg/Eug@AC beads were washed with 50 mL distilled water and the SIF was transferred to a falcon tube for 2 h mixing at a constant speed of 50 rpm. As in the SGF study, an aqueous sample was collected every 30 min for 2 h and transferred to HPLC vials [26]. At the end of this period, the SIF was removed by decantation and the remaining Alg/Eug@AC beads were stored for morphological evaluation by optical microscopy analysis.

### 2.4. Swelling Rate

In order to evaluate morphological characteristics of hydrogel beads, pre-dried hydrogel beads were accurately weighed and then immersed in simulated fluids at different pHs (pH = 1.0, 2.0, 3.0, 4.0, 7.0) [27]. After mixed with roller mixer (Australian Scientific Pty Ltd, Kotara, NSW, Australia) at 100 rpm for 2 h, hydrogel beads were separated by filtration then excessive water on beads was removed by gently wiping with soft paper [28]. The swollen beads were accurately weighed and then air-dried on filter paper for 48 h for SEM analysis. The swelling rate of the hydrogel beads was calculated using Equation (1).

(1) $$Swelling\;Rate\;\left(\%\right)=\frac{M_{s}-M_{d}}{M_{d}}\times 100$$

where *M_d_* is the initial weight of the pre-dried hydrogel beads before immersion, and *M_s_* is the weight of the hydrogel beads after immersion in solution.

### 2.5. Model-Based Analysis of Drug-Release Characteristics

To obtain a quantitative description of the drug-release behavior, experimental release data were examined using different mathematical formulations suitable for polymer-based delivery systems. Zero-order, first-order, Higuchi, and Korsmeyer–Peppas models were applied to compare the release profiles and to clarify the transport phenomena governing drug release. The mathematical expressions of the applied models are given below:

Zero-Order Kinetic Model

$$M_{t}=M_{0}-k_{0}t$$

First-Order Kinetic Model

$$(\ln M_{\infty }-M_{t})=\ln M_{\infty }-k_{1}t$$

Higuchi Kinetic Model

$$M_{t}=k_{H}\times t_{1/2}$$

Korsmeyer–Peppas Kinetic Model

MtM∞=k×tn

Here, *M_t_* represents the amount of drug released at time *t*, while M_∞_ denotes the total amount of drug released at equilibrium. The parameters *k*_0_, *k*_1_, and *k_H_* correspond to the release rate constants of the zero-order, first-order, and Higuchi models, respectively. In the Korsmeyer–Peppas equation, the constant k is related to the structural characteristics of the delivery matrix, whereas the release exponent *n* is indicative of the dominant release mechanism.

The kinetic model that best described the drug-release process was determined based on the regression coefficient (R^2^) values obtained from model fitting. The release mechanism was further evaluated using the *n* value derived from the Korsmeyer–Peppas model. Accordingly, values of *n* ≤ 0.5 were associated with Fickian diffusion, values in the range of 0.5 < *n* ≤ 0.89 indicated non-Fickian (anomalous) transport or erosion-controlled release, and values of *n* ≥ 1 corresponded to Super Case II transport [29]. Overall, the combined evaluation of these models enabled a comprehensive interpretation of the release behavior, distinguishing diffusion-controlled release from concentration-independent kinetics and release processes influenced by polymer swelling and matrix relaxation.

## 3. Results and Discussion

**Figure 1:**
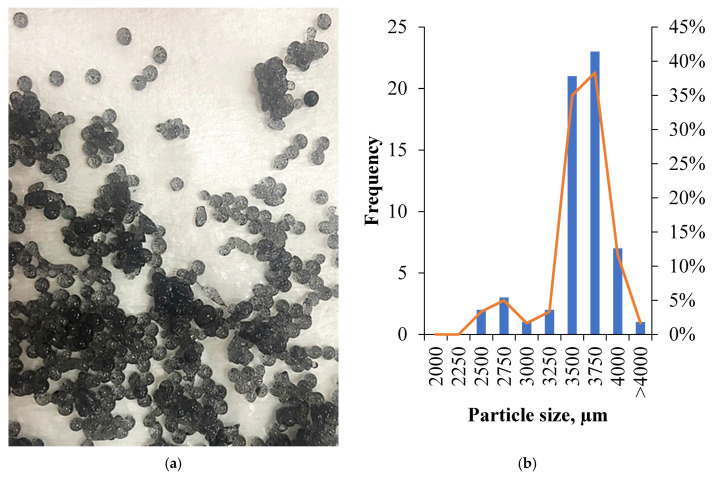
The obtained hydrogel beads (**a**) and their particle size distribution (**b**).

Figure 1 shows the hybrid microspheres composed of tea waste-derived activated carbon, sodium alginate, and eugenol, along with the average particle size distribution graph. AC from tea waste was prepared via H_3_PO_4_ activation with a yield of 30.45%, exhibiting a surface area of 87.3 m^2^/g and a 32% mesoporous structure (Figure S1). The average particle size of the hydrogel beads with spherical shape was determined to be 3401 ± 250 µm (*n* = 180 in total), based on pooled data from three independent production batches. The synthesis of sodium alginate-coated hydrogel beads was evaluated using FTIR spectroscopy, as illustrated in Figure S2, the shifts for characteristic carboxylate (–COO^−^) absorption bands were observed. The antisymmetric and symmetric stretching peaks, originally centered at 1594 cm^−1^ and 1407 cm^−1^ for pristine SA, shifted to 1588 cm^−1^ and 1415 cm^−1^ upon cross-linking, confirming the coordination between the carboxylate groups and Ca^2+^ ions [30].

**Figure 2:**
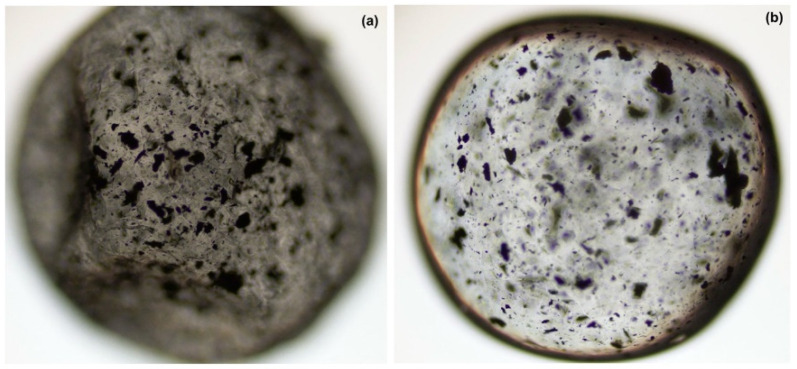
The light microscopy images of the hybrid microspheres before (**a**) and after eugenol release (**b**).

In addition, to demonstrate the stability of the microspheres, images of the hybrid microspheres were acquired using light microscopy before and after eugenol release (Figure 2). The images clearly reveal that the hybrid microspheres preserved their original shape and morphology.

**Figure 3:**
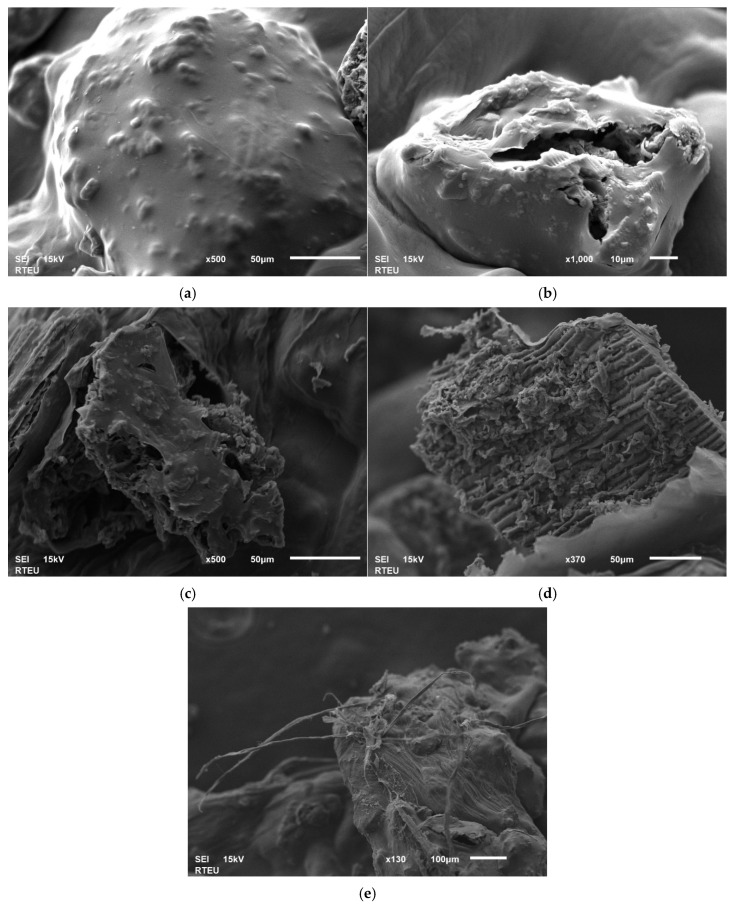
SEM images of hydrogel beads at different pHs; (**a**): pre-dried beads, (**b**): pH 1, (**c**): pH 2, (**d**): pH 3, and (**e**): pH 4.

To simulate the gastrointestinal conditions during both fasted and fed states, release studies were conducted in buffer solutions with pH values of 1.0, 2.0, 3.0, 4.0, and 7.0. As seen in Figure 3a, the pre-dried hydrogel bead exhibits a rough surface containing irregularly distributed nodule. During the swelling process in solutions with pH values ranging from 1.0 to 4.0, fragmentation from the hydrogel surface was observed (Figure 3b–d). Notably, as seen in Figure 3e, the initiation of a fibrous structure was evident at pH 4.0. Conversely, at pH 7.0, it was observed that the hydrogel beads completely lost their spherical morphological integrity and underwent a transition into a gel-like state. This morphological disintegration is quantitatively supported by the dramatic increase in swelling capacity at higher pH levels. The hydrogel beads exhibited a swelling rate of 91.12% for pH 1, 142.81% for pH 2, 148.10 for pH 3, 1350.61% for pH 4, and 1496.63% for pH 7.

**Figure 4:**
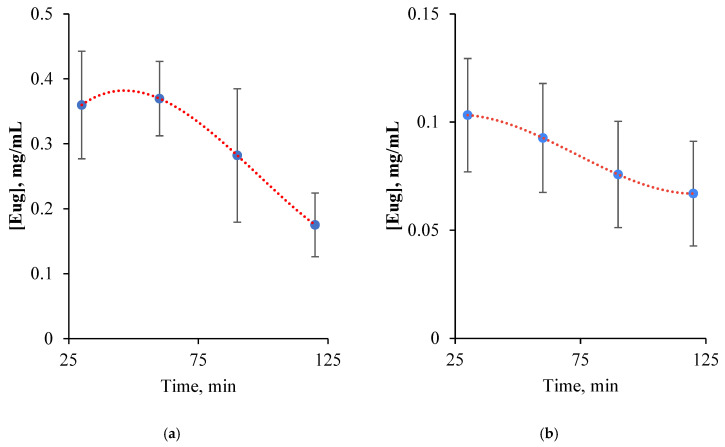
In vitro release profiles of eugenol from SGF (**a**) and SIF (**b**).

The drug adsorption and controlled release profile of hydrogel beads were monitored using a validated RP-HPLC method by Higashi and Fujii (Figure S3) [31]. A standard calibration curve was established by analyzing 0.03–1 mg/mL eugenol solutions by HPLC. A chromatographic peak was detected at 282 nm and retention time was found to be 7.84 min. Experiments for evaluation of adsorption capacity of AC were investigated 200 ppm and 400 ppm ethanolic solution of Eug. HPLC data showed that activated carbon material reached its maximum loading capacity of 113.65 ± 8.55 ppm Eug for a 200 ppm solution and 239.37 ± 7.31 ppm Eug for a 400 ppm solution within 10 min and no significant adsorption change was observed in the adsorption profile for 4 h (see Figure S4). Despite varying such parameter’s core:wall material ratio, cross linker, and extraction time, no chromatographic signal associated with Eug was detected in release experiments. Hypothesizing that the wall material presented a high diffusion barrier for low concentrations of Eug, the studies were repeated using pure Eug. HPLC data showed that the AC material reached its loading capacity of 3.37 ± 0.11 mg Eug/g hydrogel. The total released eugenol from hydrogel beads was found to be 0.694 ± 0.006 mg Eug/g hydrogel for SGF and 0.198 ± 0.002 mg Eug/g hydrogel for SIF (Figure 4). It is hypothesized that the low mesoporous volume of the activated carbon (0.018 cm^3^/g) imposes a geometric constraint on eugenol diffusion, effectively mitigating burst release and maintaining a controlled release. According to HPLC data, mean encapsulation efficiency and loading capacity of hydrogel beads were found to be 86.52 ± 2.69% and 19.23 ± 0.60% on a dry weight basis. Consistent with the previous literature, a gradual release of 20.6% of the deposited Eug was observed under acidic conditions (pH 3.0) within the first 2 h, while the drug release significantly decreased (3.5-fold) under neutral conditions within the same period [32,33].

The designed double-component drug-release system showed exceptional performance, achieving a high encapsulation efficiency of 86.52 ± 2.69%, which is highly competitive when compared to reported eugenol delivery platforms. This efficiency is higher than the 72.50% reported for pectin-modified dendritic mesoporous silica by Guo et al., and they have reported that the release rate was notably lower at pH 4.0 [34]. The encapsulation efficiency reached 68.3% in hollow silica nanorods by Zhang et al. in 72 h [35], and the approximately 60% efficiency of beeswax microcapsules with multiple preparation steps described by Peng et al. [36]. A chitosan-based controlled release system with an encapsulation efficiency of 72.63% by Cahyono et al. released > 80% eugenol in the first 15 min. They concluded that adsorbed eugenol on the surface of the particles could cause initial burst release [37]. The developed alginate/AC delivery system exhibits clear superiority over these existing platforms based on some key parameters such as a significantly higher encapsulation capacity, a more controlled and sustained release profile devoid of the premature ‘burst effect’, and a simpler, more cost-effective preparation methodology utilizing sustainable waste-derived biomass.

### Comparative Drug-Release Kinetics and Mathematical Model Fitting

**Figure 5:**
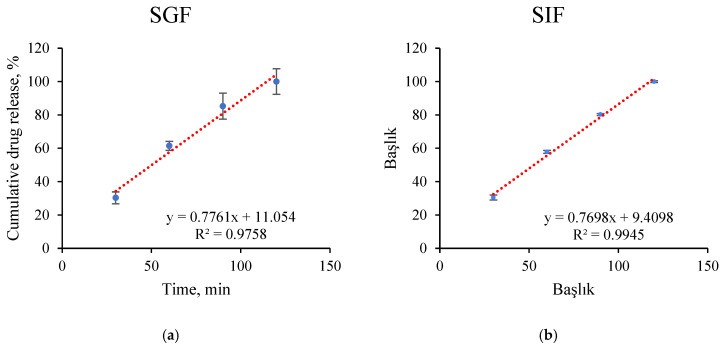

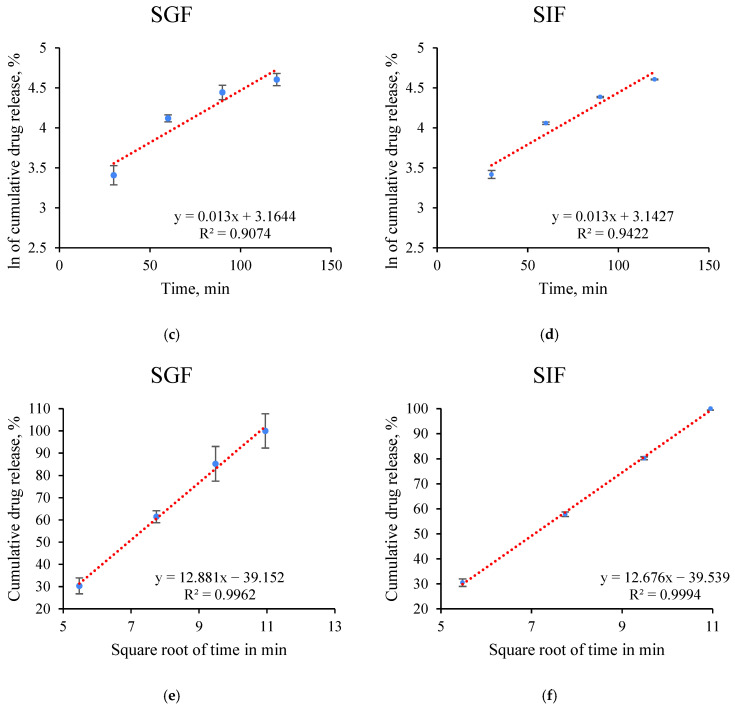
Cumulative Eug-release kinetics from the hydrogel beads under SGF and SIF conditions fitted with zero-order (**a**,**b**), first-order (**c**,**d**), and Higuchi models (**e**,**f**).

**Figure 6:**
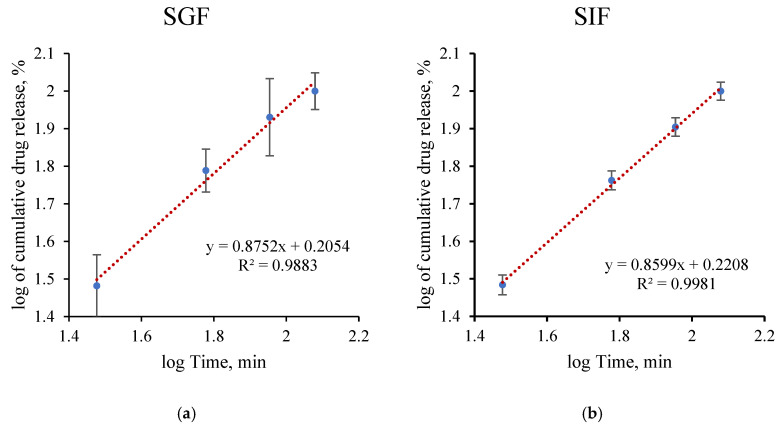
Cumulative Eug-release kinetics from the hydrogel beads under SGF (**a**) and SIF (**b**) conditions fitted with Korsmeyer–Peppas model.

Kinetic modeling of drug-release profiles is essential for elucidating the underlying release mechanisms and predicting the performance of drug delivery systems under different physiological conditions. These models provide quantitative insight into whether the release process is governed by diffusion, matrix relaxation, or a combination of multiple transport mechanisms [38,39]. Accordingly, the drug-release behavior of the developed system was investigated in simulated SGF and simulated SIF using zero-order, first-order, Higuchi, and Korsmeyer–Peppas kinetic models, as depicted in Figure 5 and Figure 6.

The corresponding kinetic parameters are summarized in Table 1. Among the evaluated models, the Higuchi model exhibited the highest correlation coefficients in both SGF (R^2^ = 0.9962) and SIF (R^2^ = 0.9994), indicating that the release process was predominantly governed by diffusion from the polymeric matrix [40].

The zero-order model also showed a good linear fit (R^2^ = 0.9758 in SGF and 0.9945 in SIF), suggesting a highly controlled and nearly constant release rate over time [41]. Moreover, the Korsmeyer–Peppas model yielded release exponent values of *n* = 0.8752 in SGF and *n* = 0.8599 in SIF, corresponding to a non-Fickian transport mechanism [39,42]. Overall, the selection of the best-fitting kinetic model was further justified by correlating the release data with the morphological changes observed in SEM analysis. As illustrated in the SEM images, the hydrogel beads underwent a significant transition from a solid-state to a gel-like form at pH 7.0, eventually losing their spherical integrity. This morphological shift directly supports the Korsmeyer–Peppas model, which exhibited the highest correlation (R^2^ > 0.98). The calculated release exponent (*n*) indicates an anomalous (non-Fickian) transport mechanism, which is physically consistent with the observed structural degradation. This suggests that the eugenol release is not merely a result of simple diffusion but is significantly governed by the matrix relaxation and swelling-induced erosion of the alginate/AC network as it transitions into a gel phase.

## 4. Conclusions

This study provides a new gastric-retentive delivery system designed to maximize the gastroprotective efficacy of eugenol while mitigating the toxicity risks associated with high dosages. It is known that low-dose administration of eugenol is critical for accelerating gastric healing. For this purpose, we designed hybrid hydrogel beads using tea waste–derived AC and sodium alginate to maintain controlled eugenol release. The specific low mesoporous architecture of the core material imposed a geometric constraint on diffusion, effectively delaying burst release. The optimized system achieved a high encapsulation efficiency of 86.52 ± 2.69% and a loading capacity of 3.37 ± 0.11 mg Eug/g hydrogel. Crucially, in vitro assays demonstrated a pH-dependent release profile suitable for local treatment; a sustained release of 0.694 ± 0.006 mg Eug/g hydrogel beads was achieved in simulated SGF, whereas release was significantly restricted to 0.198 ± 0.002 mg Eug/g hydrogel beads in simulated SIF. Consequently, this promising diffusion-controlled system offers a strategy to maintain therapeutic Eug concentrations directly at the gastric mucosa. While the current study provides critical insights into the pH-dependent release of eugenol using in vitro simulated gastric and intestinal fluids, we acknowledge that these static models do not fully replicate the complex dynamic environment of the human gastrointestinal tract. Factors such as peristaltic contractions, complex enzymatic interactions, varying transit times, and the presence of gut microbiota may influence the in vivo performance of the alginate/AC hydrogel beads.

## Figures and Tables

**Table 1:** Zero-order, first-order, Higuchi and Korsmayers–Peppas mathematical kinetics models fitting.

	Zero-Order		First-Order		Higuchi		Korsmeyer–Peppas		
	R^2^	*k*	R^2^	*k*	R^2^	*k*	R^2^	*k*	*n*
SGF	0.9758	0.7761	0.9083	0.013	0.9962	12.881	0.9883	1.605	0.8752
SIF	0.9945	0.7698	0.9423	0.013	0.9994	12.67	0.9981	1.663	0.8599

## Data Availability

The original contributions presented in this study are included in the article. Further inquiries can be directed to the corresponding author.

## Supplementary Materials

The following supporting information can be downloaded at: https://www.mdpi.com/article/10.3390/pharmaceutics18050598/s1, Figure S1: BET isotherm of the tea waste-based activated carbon, Figure S2: FTIR spectra of AC (black), sodium alginate (red), and sodium alginate coated AC (green). Figure S3: HPLC chromatograms for free eugenol (a) and after SGF treated (b). Figure S4: HPLC calibration curve for Eug (a) and adsorption profile (b).
