# Supplementary material for: Fabrication of Hybrid Alginate Hydrogel Beads Reinforced with Activated Carbon and Evaluation of Their Potential for Controlled Eugenol Release
Source: Pharmaceutics. 2026 May 14;18(5):598. doi: 10.3390/pharmaceutics18050598 (PMC13210925; doi:10.3390/pharmaceutics18050598)
Supplement: Supplementary file 1 [file pharmaceutics-18-00598-s001.zip › pharmaceutics-4256992-supplementary.pdf]

SUPPLEMENTARY DATA

Fabrication of Hybrid Alginate Hydrogel Beads Reinforced with Activated Carbon and Evaluation of Their Potential for Controlled Eugenol Release

Kaan Karaoğlu[a], Mehtap Atak[b*], Nuray Yılmaz Baran[c], and Talat Baran[c]

[a] *Department of Chemistry and Chemical Processing Technologies, Vocational School of Technical Sciences, Recep Tayyip Erdoğan University, Rize, Turkey*

[b] *Department of Medical Biochemistry, Faculty of Medicine, Recep Tayyip Erdogan University, Rize, Turkey*

[c] *Department of Chemistry, Faculty of Science and Letters, Aksaray University, 68100 Aksaray, Turkey*

Phone: +90 (464) 212 30 09

Email: mehtap.atak@erdogan.edu.tr (M. Atak)

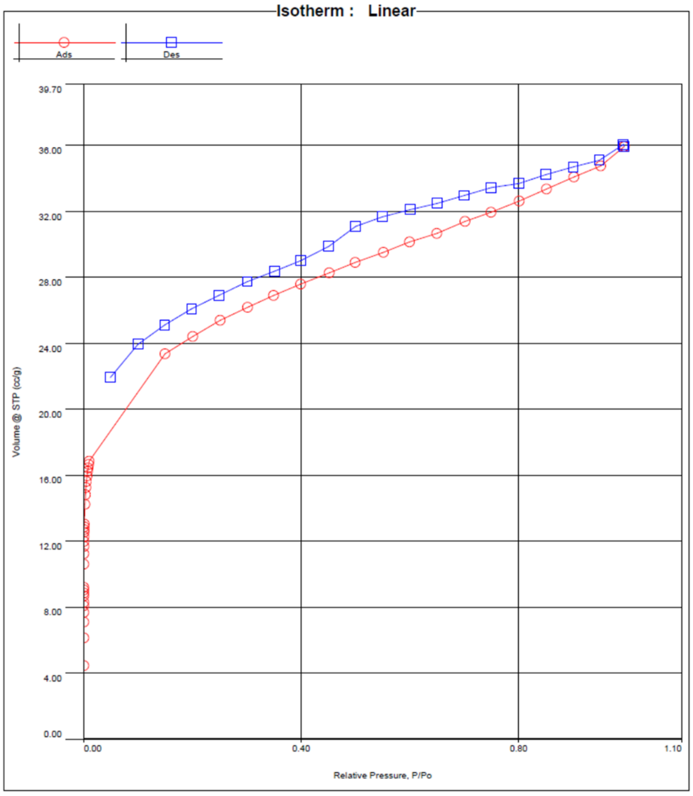

Figure S1. BET isotherm of the tea waste-based activated carbon.

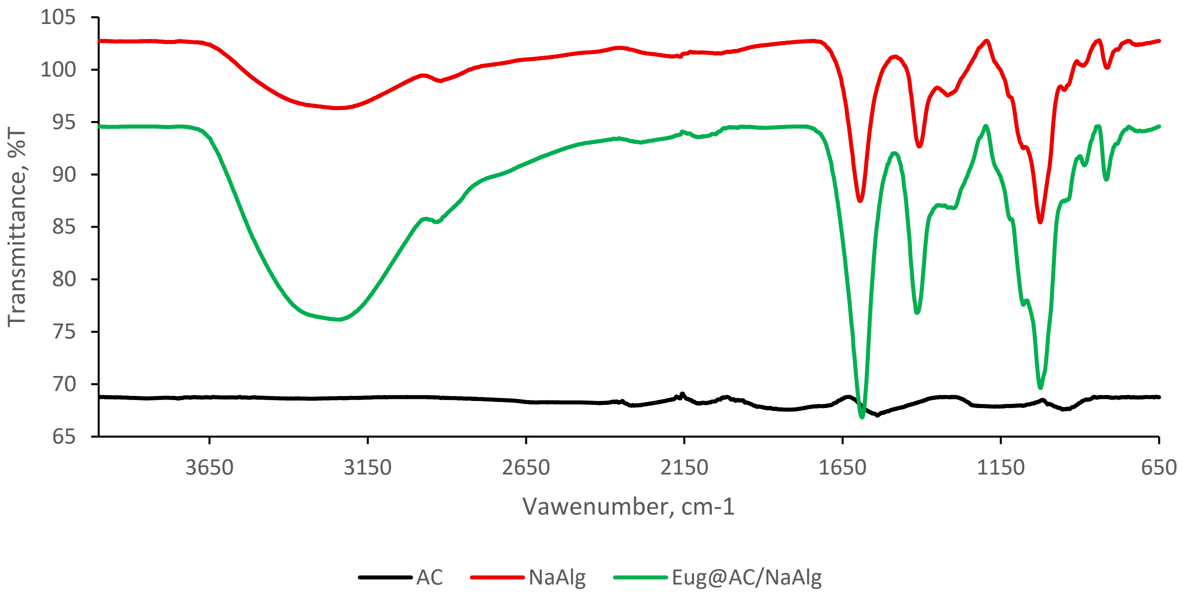

Figure S2. FTIR spectra of AC (black), sodium alginate (red), and sodium alginate coated AC (green).

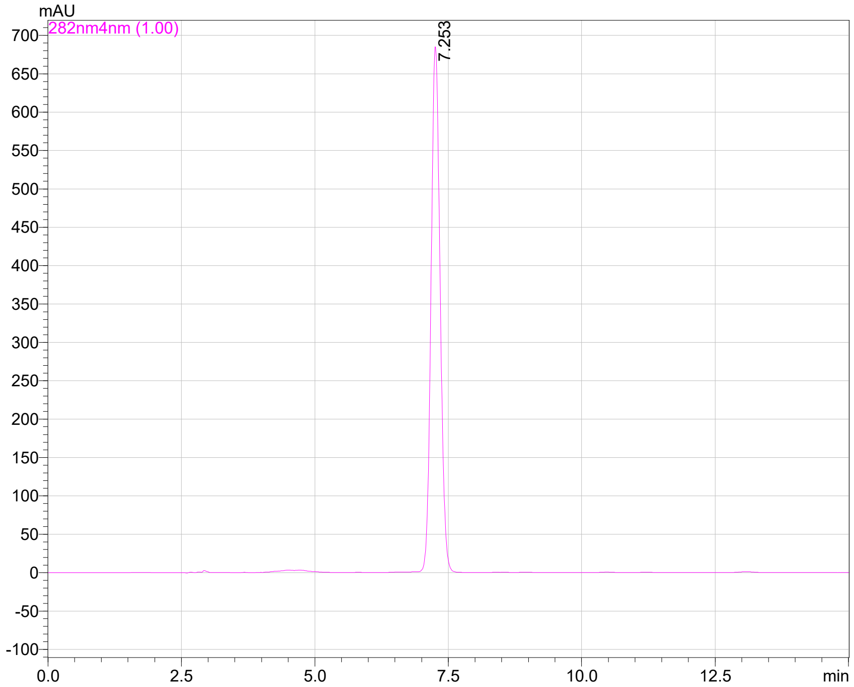

a

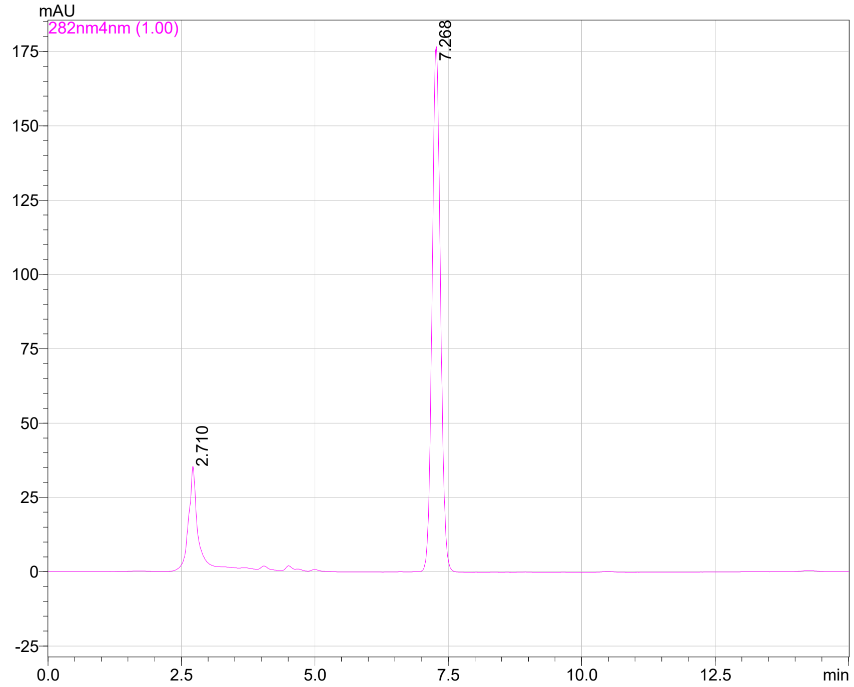

b

Figure S3. HPLC chromatograms for free eugenol (a) and after SGF treated (b).

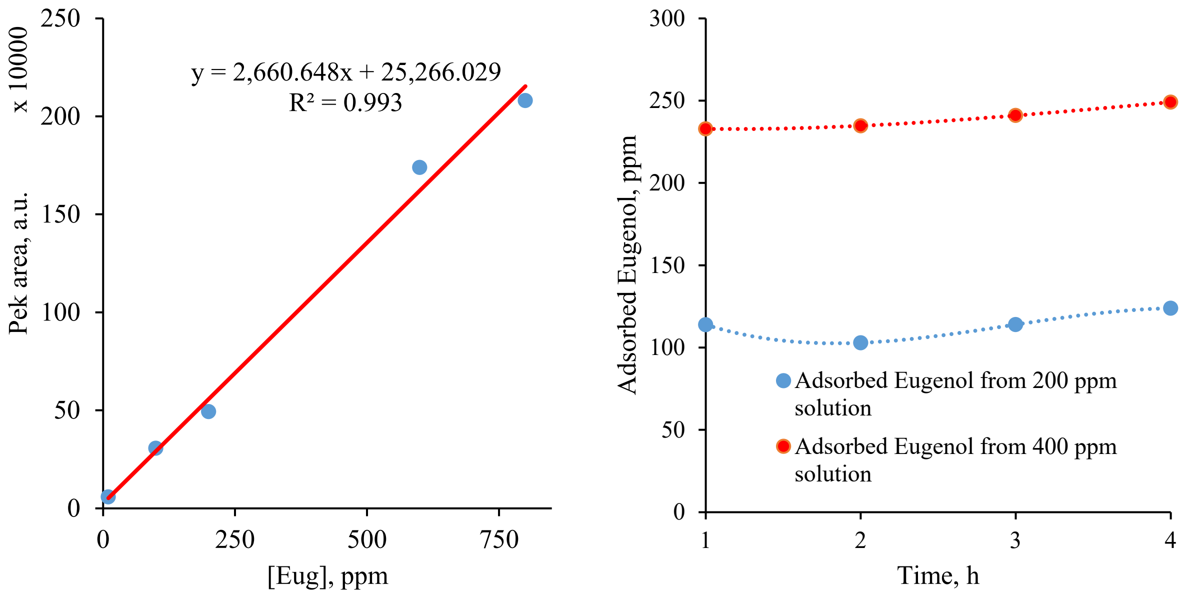

a b

Figure S4. HPLC calibration curve for Eug (a) and adsorption profile (b).
